# Comparison of the extraperitoneal and transperitoneal routes for permanent colostomy: a meta-analysis with RCTs and systematic review

**DOI:** 10.1186/s12957-022-02547-9

**Published:** 2022-03-12

**Authors:** Jinlong Luo, Dujanand Singh, Faqiang Zhang, Xinting Yang, Xiaoying Zha, Huaiwu Jiang, Lie Yang, Hua Yang

**Affiliations:** 1 Department of Colorectal Anal Surgery, Zigong Fourth People’s Hospital, Zigong, Sichuan China; 2 Laboratory of Digestive Surgery, West China Hospital and State Key Laboratory of Biotherapy, Sichuan University, Chengdu, Sichuan China; 3 Department of Wound Care Center, West China Hospital, Chengdu, Sichuan China; 4 Department of Wound Care Center, Department of Colorectal Anal Surgery, Zigong Fourth Hospital, Zigong, Sichuan China; 5 Department of General Surgery, Sichuan Mianyang 404 Hospital, Mianyang, Sichuan China

**Keywords:** Meta-analysis, Extraperitoneal, Transperitoneal, Colostomy, Complication, Parastomal hernia, RCT

## Abstract

**Aim:**

To assess the efficacy of extraperitoneal colostomy (EPC) in preventing stoma-related complications.

**Background:**

Transperitoneal colostomy (TPC) is a widely used surgical approach. However, TPCs have been reported to have increased risks of stoma-related complications, such as parastomal hernias, stomal retraction, and stomal prolapse. The purpose of EPC is to reduce these complications. However, there is still a lack of evidence-based studies.

**Materials and methods:**

MEDLINE, EMBASE, Web of Science, Scopus, MOOSE, PubMed, Google Scholar, Baidu Scholar, and the Cochrane Library were searched to conduct a systematic review and meta-analysis with RCTs. The meta-analysis was performed with RevMan 5.4 software.

**Results:**

This study included 5 eligible RCTs. Compared with the TPC group, the EPC group had lower incidence rates of parastomal hernias (RR, 0.14; 95% CI, 0.04–0.52, *P* = 0.003, *I*^2^ = 0%) and stomatal prolapse (RR, 0.27; 95% CI, 0.08–0.95, *P* = 0.04, *I*^2^ = 0%), but a higher rate of defecation sensation (RR, 3.51; 95% CI, 2.47–5.0, *P* < 0.00001, *I*^2^ = 37%). No statistically significant differences were observed in stoma retraction, colostomy construction time, stoma ischemia, or stoma necrosis.

**Conclusion:**

Extraperitoneal colostomies are associated with lower rates of postoperative complications than transperitoneal colostomies. A randomized controlled trial meta-analysis found that permanent colostomies after abdominoperineal resection resulted in better outcomes.

## Background

Abdominoperineal resection with a permanent colostomy is a standard procedure for patients undergoing surgery for low rectal cancer [1]. Traditionally, a permanent stoma is constructed using a transperitoneal colostomy (TPC). However, TPCs are associated with an increased risk of stoma-related complications, such as parastomal hernias, stomal retractions, stomal prolapses, and stoma-related blood supply disorders [2]. The overall incidence of parastomal hernia has been reported to be up to 50% or higher in long-term follow-ups [3]. Although many complications are asymptomatic, they can cause discomfort or even life-threatening problems [4]. The extraperitoneal approach to stoma construction, which was first reported by Goligher, has been shown to decrease the rate of parastomal hernias and small bowel obstructions [5]. Furthermore, Hamada et al. developed a laparoscopic technique for extraperitoneal colostomies and found an effective way to reduce the incidence of parastomal hernias [6]. Jin et al. [7] reported fewer long-term stoma-related complications in the EPC group. However, subsequent studies have yielded inconsistent results. The efficacy of EPC in permanent colostomies remains controversial in the available studies.

Therefore, the goal of this meta-analysis was to compare the efficacy of extraperitoneal versus transperitoneal colostomy with random controlled trials (RCTs).

## Materials and methods

All aspects of the Preferred Items for Reporting of Systematic Reviews and Meta-Analyses (PRISMA) [8] and Meta-Analysis of Observational Studies in Epidemiology (MOOSE) [9] guidelines were followed.

### Search strategy and data collection

A systematic search of the main medical databases, including MEDLINE, EMBASE, Web of Science, Scopus, MOOSE, PubMed, Google Scholar, Baidu Scholar, and the Cochrane Library, was performed using the following interchangeable terms: “extraperitoneal,” “transperitoneal,” “intraperitoneal,” “rectal cancer,” “laparoscopic abdominoperineal resection,” “parastomal hernia,” “colostomy,” “sigmoidoscopy,” and “stoma or ostomy,” as well as related medical subject headings. We conducted a comprehensive search for all RCTs that have been published to date. We manually searched the references of the retrieved articles and identified additional published articles. The study protocol was registered in the International Prospective Register of Systematic Reviews database (ID: CRD42021271251).

Two authors (Jinlong Luo and Dujanand Singh) separately read the titles of the identified references and eliminated irrelevant studies. The final evaluation was performed independently based upon an examination of the entire text. The inclusion criteria were RCT studies with fully published papers that included the outcomes of both extraperitoneal colostomies and transperitoneal colostomies. Two researchers independently extracted topic-related studies that evaluated at least one of these outcomes: parastomal hernias, stomal prolapses, stomal retractions, stomal ischemia and necrosis, colostomy construction time, and defecation sensation data. We resolved conflicts of interest by discussing and consulting with a third reviewer.

### Assessment of study quality

The possible risk of bias was assessed using the Cochrane Collaboration’s risk for bias assessment tool [10]. Two reviewers evaluated the quality of the studies, and disagreements were discussed with a third reviewer.

### Statistical analysis

RevMan software (version 5.4; The Nordic Cochrane Centre, Copenhagen, Denmark) was used to perform the meta-analysis. Risk ratios (RRs) and 95% confidence intervals (CIs) were used to compare the following variables: parastomal hernias, stomatal prolapse, stoma retraction, stoma ischemia, stoma necrosis, and defecation sensation. Mean differences (MD) were used to analyze the colostomy construction time. The *I*^2^ statistic was used to assess statistical heterogeneity, and we considered an *I*^2^ of 0–40% to be essentially unimportant, 30–60% to represent moderate heterogeneity, 50–90% to represent substantial heterogeneity, and 75–100% to represent considerable heterogeneity. A random-effects model was used if clinical heterogeneity was observed during the study, while a fixed-effects model was used if the observed heterogeneity was low. We intended to investigate the heterogeneity and perform a subgroup analysis if appropriate. The significance level was set at *P* = 0.05 in both models.

## Results

### Description of eligible studies

**Fig. 1:**
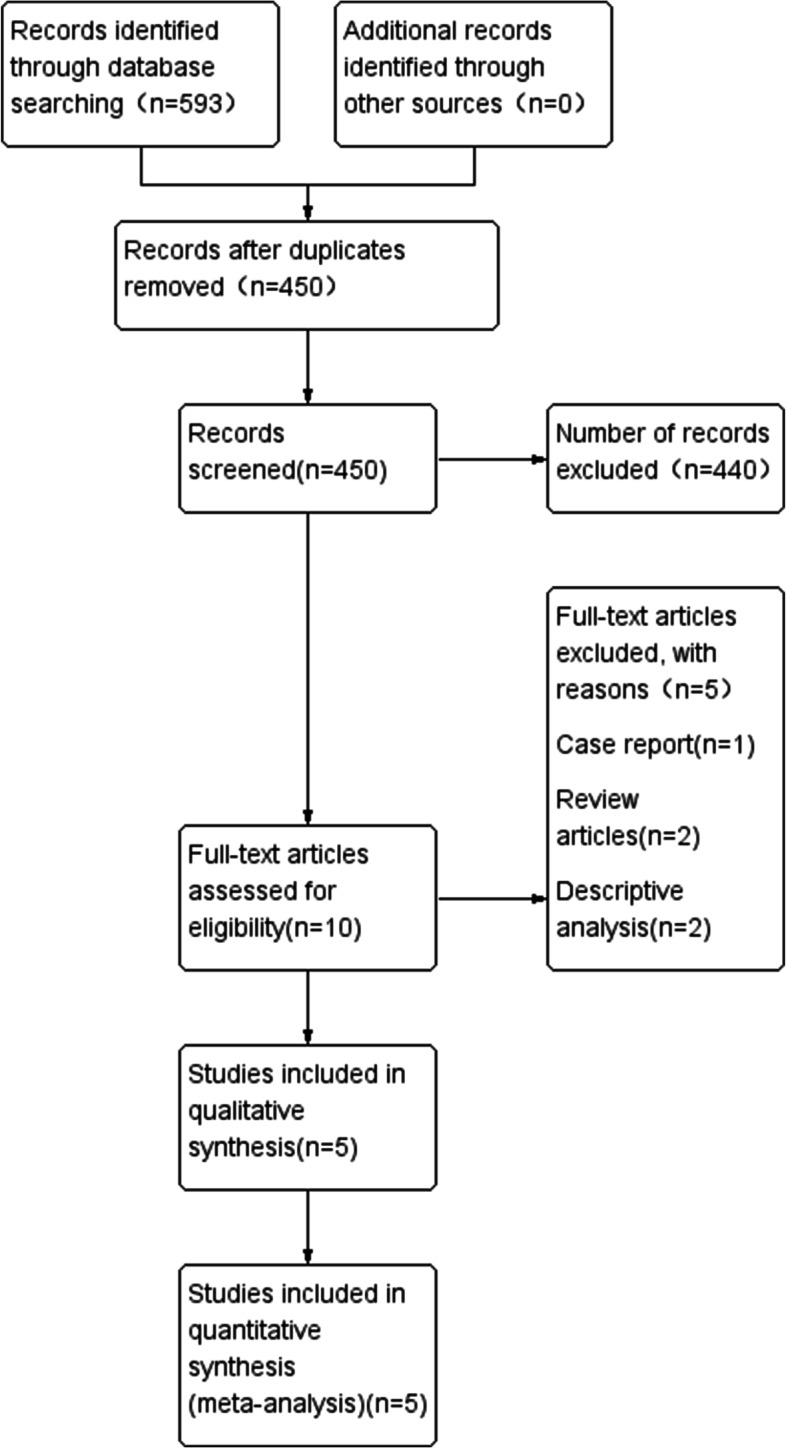
Flow diagram of studied identified, included and excluded

**Fig. 2:**
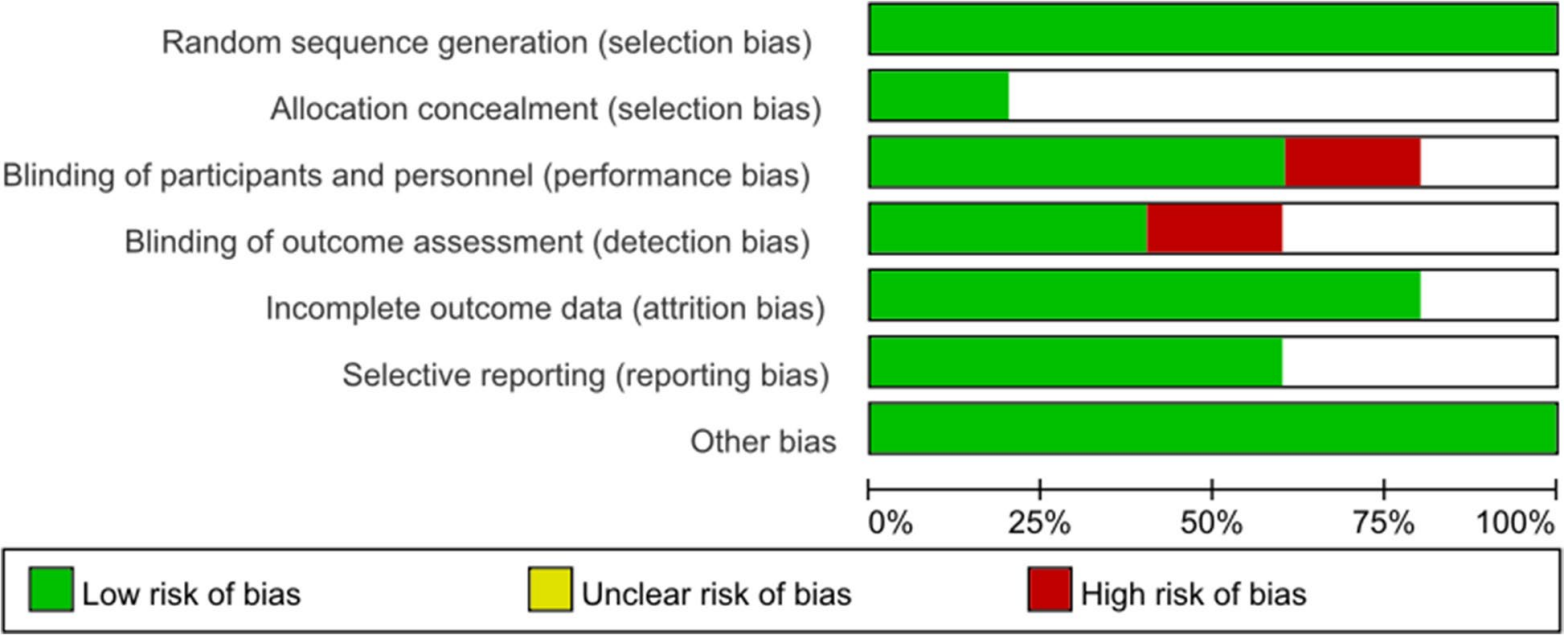
Summary of risk of bias assessment

We identified 593 studies from the database search and other sources. A flow diagram was used to identify eligible studies (Fig. 1). A total of 5 articles were selected that included 417 patients (211 patients in the extraperitoneal group and 207 patients in the transperitoneal group). Among those patients, 311 patients underwent laparoscopic surgery, while 106 patients underwent open surgery. All patients were from China. The basic study characteristics are summarized in Table 1. The risk bias assessment is presented in Fig. 2.

**Table 1:** Characteristics of the studies included in the meta-analysis

Reference	Primary surgery	Follow-up, months	Patients (EPC/TPC)	Parastomal hernia (EPC/TPC)	Stoma prolapse (EPC/TPC)	Stoma retraction (EPC/TPC)	Stoma ischemia and necrosis (EPC/TPC)	Defecation sense (EPC/TPC)	Operating time, min (EPC/TPC)	
Wu et al. 2017 [11]	OAPR	12	53/53	0/3	0/3	0/1	1/1	–	22.8 ± 2.4/16.4 ± 1.5	
Ye et al. 2014 [12]	LAPR	6–36	41/40	0/3	1/1	0/1	1/2	38/11	–	
Zhou et al. 2016 [13]	LAPR	1–12	33/34	0/4	0/2	0/2	0/2	11/6	13.4 ± 1.7/16.4 ± 2.6	
Jin et al. 2014[14]	LAPR	12–24	18/18	0/2	0/1	0/0	1/1	–	25.3 ± 8.5/14.7 ± 6.4	
Dong et al. 2012 [15]	LAPR	6–60	66/62	0/5	0/2	1/3	1/1	51/10	21.3 ± 3.5/30.4 ± 4.2	

*OAPR* open abdominoperineal resection, *LAPR* laparoscopic abdominoperineal resection, *EPC* extraperineal colostomy, *TPC* transperineal colostomy, *Operating time* colostomy construction time

### Parastomal hernia

**Fig. 3:**
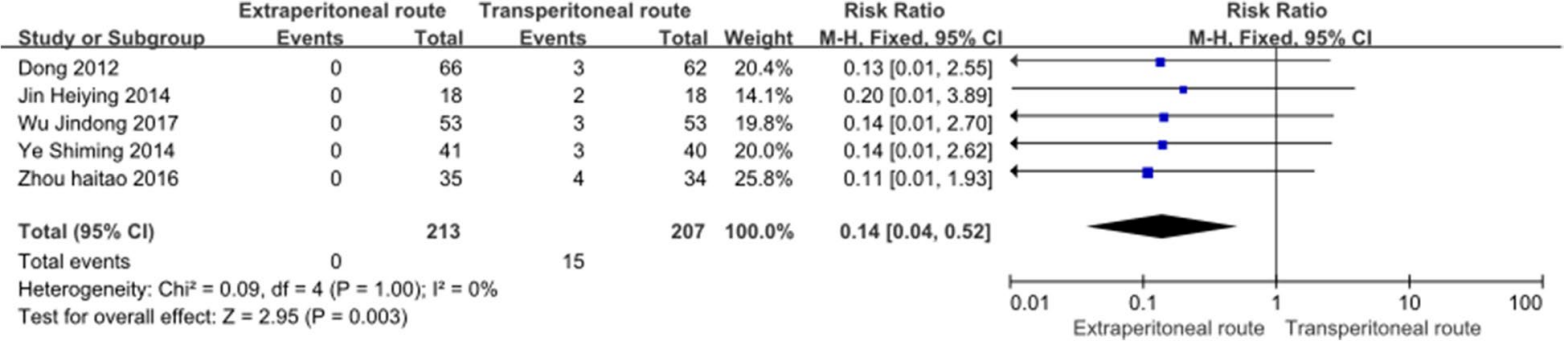
Forest plot of parastomal hernia

All five included studies [11–15], which included a total of 420 patients (213 patients with EPC and 207 patients with TPC), reported parastomal hernias. In our analysis (Fig. 3), the rate of parastomal hernia was statistically lower when the extraperitoneal route and a laparoscopic approach were used (RR, 0.14; 95% CI, 0.04–0.52, *P* = 0.003, *I*^2^ = 0%).

### Stomal prolapse

**Fig. 4:**
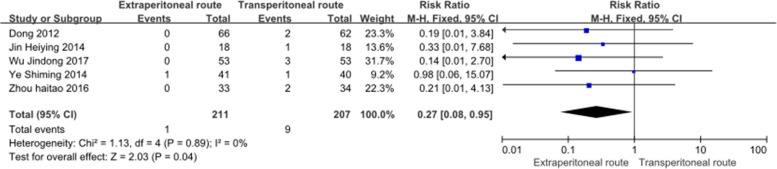
Forest plot of stoma prolapse

Five studies [11–15] with 418 patients (211 patients with EPC and 207 patients with TPC) reported stomal retraction (Fig. 4). The analysis revealed that the extraperitoneal route was associated with a lower incidence of stomal prolapse than the transperitoneal route (RR, 0.27; 95% CI, 0.08–0.95, *P* = 0.04, *I*^2^ = 0%).

### Stomal retraction

**Fig. 5:**
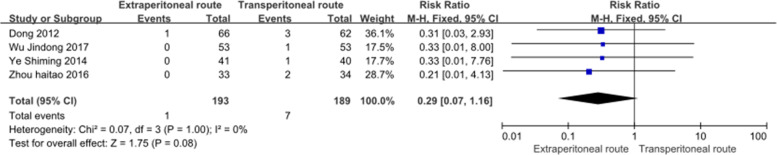
Forest plot of stoma retractions

Four studies [11–13, 15] reported a lower rate of stomal retraction in the EPC group than in the TPC group, but the difference was not statistically significant (RR, 0.29; 95% CI, 0.07–1.16, *P* = 0.08, *I*^2^ = 0%) (Fig. 5).

### Stomal ischemia and necrosis

**Fig. 6:**
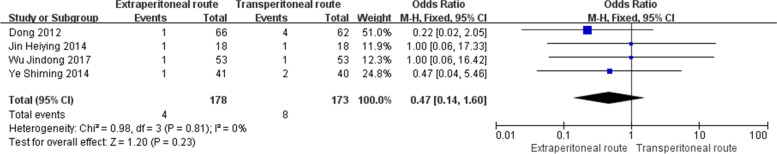
Forest plot of stoma ischemia and necrosis

Among the four included studies, two papers reported stomal ischemia [11, 14, 15]. One paper reported stoma necrosis [13], and one reported compromised stoma blood supply [12]. Because avascular ischemia and necrosis are both caused by blood supply disorders, we combined the data. The pooled data showed no significant difference (RR, 0.47; 95% CI, 0.14–1.60, *P* = 0.23, *I*^2^ = 0%) (Fig. 6).

### Sensation of defecation

**Fig. 7:**
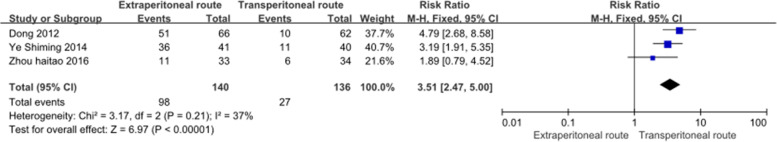
Forest plot of defecation sensation

Three studies [12, 13, 15] reported issues with defecation sensation. All these studies found a higher sensation of defecation in the extraperitoneal group than in the transperitoneal group, and this difference was statistically significant (RR, 3.51; 95% CI, 2.47–5.0, *P* < 0.00001). A random-effects model was used to validate the results (*I*^2^ = 78%) (Fig. 7). The heterogeneity decreased after the study by Zhou [13] was excluded, and we again found that the extraperitoneal route was associated with a higher rate of sensation than the transperitoneal route (RR, 18.15; 95% CI, 8.95–36.78, *P* < 0.00001).

### Colostomy construction time

**Fig. 8:**
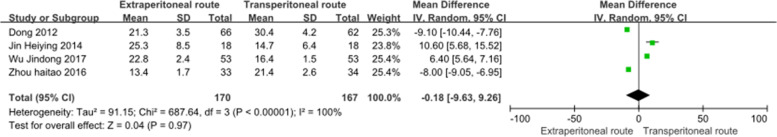
Forest plot of colostomy construction time

Four studies [11, 13–15] reported on the colostomy construction time. Two studies [13, 15] found a statistically significant reduction in colostomy construction in the extraperitoneal group. One study [14] reported a statistically significant longer colostomy construction time in the extraperitoneal group. One study [12] found no significant difference between the two groups. Our analysis showed that there was no statistically significant difference in construction time (MD, -0.18; 95% CI, -9.63–9.26, *P* = 0.97, *I*^2^ = 100%) (Fig. 8).

## Discussion

Stoma-related complications include parastomal hernias, stomal prolapse, stomal retraction, stomal ischemia, and stomal necrosis. These are issues that are difficult to avoid [13, 16, 17]. EPCs are designed to reduce the incidence of stoma-related complications. Previous studies have suggested that extraperitoneal colostomies have many benefits with fewer complications [6, 18]. However, there is still a lack of evidence-based medical data. We included five RCTs to evaluate two distinct operative methods for creating permanent colostomies and assessing the effectiveness of colostomies.

### Parastomal hernia

Parastomal hernias are the most common complication following permanent stoma formation [16]. The incidence of parastomal hernias varies greatly in the literature; they develop most frequently within the first 2 years after stoma formation, with an incidence of up to 50%, and the risk persists for more than 20 years [17, 18]. Most parastomal hernias are asymptomatic and do not require surgical treatment. However, there are still some life-threatening complications, such as strangulation, perforation, and obstruction. Although several techniques have been reported for preventing parastomal hernias, the results have been mixed [18–20]. Therefore, preventing parastomal hernias is the best option. EPC has been considered as a solution for decreasing the rate of parastomal hernias [21]. The meta-analysis of 1048 patients showed that EPC was associated with a lower rate of parastomal hernia [22]. This is consistent with the majority of previous evidence [21, 23, 24].

Our study also showed that the incidence of parastomal hernia was lower with the extraperitoneal route than the transperitoneal route. The possible reasons for this include the lateral space between the colon and the abdominal wall caused by the surgery. pulled out through the extraperitoneal space, which is an effective method of avoiding this lateral space. In addition, the transperitoneal route provides additional coverage of the lateral peritoneal flap; to some extent, this may strengthen the abdominal wall, while the force on the abdominal wall is more evenly distributed with the lateral peritoneal flap [25]. Furthermore, the larger contact surface between the colon and peritoneum increases direct friction, decreasing the risk of hernias [26].

Thus, our analysis showed a good result for EPC. There are still some shortcomings that cannot be ignored. First, there are currently no agreed-upon diagnostic criteria for parastomal hernias. Second, the published articles did not discuss the diagnostic criteria. Third, all the included articles were small, single-center studies.

### Stomal prolapse

Stomal prolapse is a common complication of stoma formation. The incidence rate varies and depends on systematic and long-term follow-up. The prevalence of stomal prolapse varies by type and ranges from 2 to 22% [27, 28]. There is disagreement about the effect of an extraperitoneal stoma on stomal prolapse. When comparing the meta-analysis with EPC and TPC, Lian et al. [29] found no significant difference in the prolapse rates (3.4% vs. 5.7%) (OR = 0.61, *P* = 0.38). Kroese et al. [22] reported a decreased incidence of prolapse in EPC in the meta-analysis, and the overall incidence of stoma prolapse was 2 out of 185 (1.1%) after extraperitoneal construction, as opposed to 13 out of 179 (7.3%) after transperitoneal construction (RR = 0.21, *P* = 0.01). Our results showed that EPC had a statistically significant lower rate of prolapse than TPC. Some surgeons believe that intra-abdominal stoma fixation can prevent this complication. Goligher [5] hypothesized that EPC may reduce the incidence of prolapse because the bowel is better fixed by an oblique exit from the abdomen. The oblique tunnel of the bowel through the abdominal wall reduces the likelihood of prolapse by increasing friction and adhesion.

### Stomal retraction

The overall incidence of stomal retraction ranges from 1.4 to 9%. Retraction is often associated with additional complications, including leakage, mucocutaneous separation, peristomal skin, and peristomal abscess, all of which have a negative impact on the quality of life of patients [30, 31]. Our statistical analysis showed no significant difference in stomal retraction between the EPC and TPC groups (0.51% vs. 3.70%, *P* = 0.08). The most common cause of stomal retraction is excessive tension on the stoma, which is usually the result of inadequate mobilization. This could be solved by adequate sigmoid mobilization, descending colostomies, and splenic flexure [5, 32, 33].

### Stoma ischemia and necrosis

Stomal ischemia and necrosis are early complications that have been reported in up to 20% of ostomy patients [34]. The published data showed no difference in stomal ischemia and necrosis between the EPC and TPC groups [35]. The major cause of ischemia and necrosis is a reduction in blood supply, which is mainly caused by excessive trimming of epiploic fat and the mesentery [36]. However, the statistical analysis revealed no significant difference between the EPC and TPC groups (2.24% vs. 4.62%, *P* = 0.23). EPC does not appear to increase the risk of blood supply disorders.

### Sensation of defecation

The sensation of defecation was comparatively more common in the extraperitoneal group than the transperitoneal group (*P* < 0.00001). The sensation of defecation is a new reflex that may develop when feces pass through the colon covered by the peritoneum. The abundant nerve endings in the parietal peritoneum are stimulated by the passage of feces [15]. Following EPC, contact between the sigmoid colon and the peritoneum is established, resulting in varying levels of defecation control through the contraction of abdominal muscles. To some extent, EPC has been shown to improve the quality of life of patients.

### Colostomy construction time

Colostomy construction time reflects a surgeon’s skill level. Wang et al. [37] analyzed 231 patients and found that the average time was 19 min in the extraperitoneal group and 27 min in the transperitoneal group (*P* < 0. 001). Zhang et al. [38] found that there was no significant difference between the groups. The time required to create the extraperitoneal stoma was mainly spent in creating the extraperitoneal tunnel, and less time was required for suture and fixation. Our analysis showed a statistically equivalent result with high heterogeneity (*I*^2^ = 100%). Compared with TPC, EPC did not significantly increase the colostomy construction time. However, more studies are needed to identify standard surgical procedures.

The main limitation of our study was the small number of single-center trials. High-quality, multicenter randomized controlled trials with a large number of patients are needed to perform further analysis.

## Conclusion

In conclusion, based on the evidence, permanent colostomy via an extraperitoneal route had more advantages than transperitoneal colostomy.

## Data Availability

All data generated or analyzed during this study are included in this published article.

## Abbreviations

EPC: Extraperitoneal colostomy
TPC: Transperitoneal colostomy
OAPR: Open abdominoperineal resection
LAPR: Laparoscopic abdominoperineal resection
